# What makes an effective Quality Improvement Manager? A qualitative study in the New Zealand Health System

**DOI:** 10.1186/s12913-021-07433-w

**Published:** 2022-01-10

**Authors:** Adeel Akmal, Nataliya Podgorodnichenko, Tim Stokes, Jeff Foote, Richard Greatbanks, Robin Gauld

**Affiliations:** 1 Department of General Practice and Rural Health, Dunedin School of Medicine, University of Otago, Dunedin, New Zealand; 2 Centre for Health Systems and Technology, University of Otago, Dunedin, New Zealand; 3 DBA, Otago Business School, University of Otago, Dunedin, New Zealand; 4 Department of Management, Otago Business School, University of Otago, Dunedin, New Zealand; 5 Dean’s Office, Otago Business School, University of Otago, Dunedin, New Zealand

**Keywords:** Quality improvement, Health policy, Health services research, Quality improvement managers, Qualitative research, New Zealand

## Abstract

**Purpose:**

Quality improvement is an international priority, and health organisations invest heavily in this endeavour. Little, however, is known of the role and perspectives of Quality Improvement Managers who are responsible for quality improvement implementation. We explored the quality improvement managers’ accounts of what competencies and qualities they require to achieve day-to-day and long-term quality improvement objectives.

**Design:**

Qualitative exploratory design using an interpretivist approach with semi-structured interviews analysed thematically.

Setting and participants.

Interviews were conducted with 56 quality improvement managers from 15 (out of 20) New Zealand District Health Boards. Participants were divided into two groups: traditional and clinical quality improvement managers. The former group consisted of those with formal quality improvement education—typically operations managers or process engineers. The latter group was represented by clinical staff—physicians and nurses—who received on-the-job training.

**Results:**

Three themes were identified: quality improvement expertise, leadership competencies and interpersonal competencies. Effective quality improvement managers require quality improvement experience and expertise in healthcare environments. They require leadership competencies including sense-giving, taking a long-term view and systems thinking. They also require interpersonal competencies including approachability, trustworthiness and supportiveness. Traditional and clinical quality improvement managers attributed different value to these characteristics with traditional quality improvement managers emphasising leadership competencies and interpersonal skills more than clinical quality improvement managers.

**Conclusions:**

We differentiate between traditional and clinical quality improvement managers, and suggest how both groups can be better prepared to be effective in their roles. Both groups require a comprehensive socialisation and training process designed to meet specific learning needs.

**Supplementary Information:**

The online version contains supplementary material available at 10.1186/s12913-021-07433-w.

## Background

Quality Improvement (QI) methods—lean thinking, six sigma, model for improvement (espoused and adopted by the Institute for Healthcare Improvement), co-design and the like—are indispensable in ensuring safe, effective and timely patient care [1–6]. Lean thinking and six sigma have their origins in manufacturing industry, where they have become the modus operandi for organisations chasing a defect-free quality process while also aiming to achieve customer satisfaction [7–9]. On the other hand, models for improvement and co-design methods have been developed specifically for healthcare systems [2, 10, 11].

In pursuing QI, Health Organisations (HOs) invest heavily in training clinical and managerial staff to use these methods in the hope of closing QI-related gaps in patient care [12–16]. QI implementation is complex—it spans over multiple organizational levels requiring active participation of employees and managers, and involve improvements and modifications of the underlying care processes. However, examples of truly system-wide QI implementations are still uncommon [9, 17, 18]. The majority of QI implementations tend to be small in scope, being limited to single HO departments [19]. They also remain focused on tool-based QI, i.e. implementation of a few simplified QI tools in a single value-stream or process without exploring and implementing the soft side of QI: QI culture based on value for customers (patients, families and HO employees), empowerment and continuous improvement [20–22]. Thus they often fail to bring about desired improvements [9, 10, 18, 22–26]. Previous research has explored the reasons for QI failure by focusing on different QI initiatives and methodologies as well as their compatibility with the logic of healthcare [23, 24, 26–28]. However, the perspectives of Quality Improvement Managers (QIMs) who have responsibility for QI implementation has not been well-studied to date [29, 30].

QIMs play an essential role in initiating and driving QI transformations and must be prepared for this role as QI initiatives are ‘highly contested’ by healthcare stakeholders [31]. Indeed, resistance to QI initiatives in HOs is well-documented in the literature [20, 22, 31]. Clinical staff adhere to medical professionalism and care logics which are often at odds with the managerial logic underpinning QI [12, 32, 33]. This situation requires QIMs to serve as a linchpin between medical and managerial interests and practices by using strategies to minimise the clash between each logic [28]. Thus, the competencies and qualities of QIMs, which enable them to navigate the social complexity of HOs are a key success factor in QI implementation [29]. However, there is limited research devoted to understanding the competencies and qualities of successful QIMs [13, 29, 34]. This paucity of research essentially precludes our understanding of the requirements to QIMs’ competencies and how they could be developed to make QIMs successful in their roles in HOs. We posit that research in this area will balance the scales in QI research—between organisational and individual level—and focus attention of researchers and practitioners on the individual antecedents of QI *performance.*

This study aims to address this gap by exploring QIMs’ accounts of the competencies and qualities they require in accomplishing the everyday and long-term objectives of QI in their organisations.

## Methods

### Study design and sample

This study adopted an interpretivist approach giving central importance to participants’ experiences and meanings as these guide their behaviour and draw out the context in which participants operate [35, 36]. Aligned with the interpretivist approach, a qualitative exploratory design is used to investigate participants’ viewpoints, experiences and attitudes [37, 38].

To provide a nuanced understanding of QIM competencies and skills, we compared the perspectives of two different groups of QIMs present specifically in the healthcare sector, something previous research has not focused upon as it relied on manufacturing and other industries [29, 34]. The first group consists of those with formal QI education and training—typically former operations managers or process engineers who have joined HOs—we call them traditional QIMs. The second group is represented by clinical staff—physicians and nurses—who (in most cases) received informal on-the-job QI training or were sent to an external institute to get QI training during their employment with their respective HOs. We refer to them as clinical QIMs. The reason for comparing these two participant groups is their different professional backgrounds creates different experiences and special developmental needs required to be an effective QIM.

Participants were recruited from 15 New Zealand (NZ) District Health Boards (DHBs), regional HOs tasked with planning, funding and delivery of the country’s predominantly publicly-funded healthcare services. DHBs are responsible for healthcare quality and undertake service improvement to improve patient safety and reduce operating costs and inefficiencies [39]. QIMs, in their respective DHBs, are tasked with designing, implementing and supporting QI initiatives within and across the clinical and non-clinical operations and directorates of the organisations [19].

To gain a thorough understanding of the research problem we used a purposive sampling approach [40–42]. Participants were invited to take part in the study based on several key criteria: (1) they were part of the QI directorate/division in their respective DHB and active in the implementation of QI initiatives; (2) they had previous QI training; (3) they had worked with this particular DHB for more than two years; (4) QI background (traditional or clinical). Parameters such as their age, gender, ethnic background were not considered to have any effect on our research question, and hence, they were not considered in our participant selection criteria.

To identify the points of contact, a member of the senior leadership team from each DHB was approached for either an interview or recommendation of the most appropriate participant from their DHBs. All recommended participants were then contacted directly via email. Participants were provided with an information sheet that explained the research objectives and study design along with a research participation consent form. DHBs and their quality directorates tend to be small, and QIMs across the sector know each other quite well. Therefore, protecting participant confidentiality was very important and is reflected in our presentation of participant characteristics (Table 1).

**Table 1:** Participant Information

Participant Information	
QIMs	
Traditional QIMs	20
Clinical QIMs (22 nurses and 14 medical practitioners)	36
DHB Type	
Metropolitan DHBs	34
Rural and Provincial DHBs	22
Years of Experience	
2 to 5 (early-career QIMs)	23
> 5	33

Metropolitan DHBs are where at least 50% of the population is living in metropolitan and urban areas. Rural DHBs have scattered populations throughout their jurisdiction

### Data collection and analysis

The data were collected through semi-structured interviews between June 2017 and December 2018. The semi-structured nature of interviews allowed the research team to stay open to the participants’ answers, and develop new questions to pursue new themes emerging in the interview while ensuring that all the key topics related to the research questions are covered by all participants [43]. An interview schedule was developed, and pilot tested with six QIMs from private HOs operating in the country. The data from these interviews were not included in the final analysis and were only used to improve the interview guidelines to ensure the research validity and ability to address research questions [44]. The questions included in the final schedule (Supplementary File 1) included the nature of QI and its adoption in their respective HOs, key job responsibilities of QIMs, requirements of their jobs, their understanding of success factors allowing them to accomplish their everyday and long-term objectives, and challenges they faced trying to attain their goals. Such questions allowed us to gain an understanding of the competencies and qualities they require in accomplishing the everyday and long-term objectives of QI in their organisations. In this paper we focus our analysis on the responses to the interview questions that relate to the competencies and qualities QIMs require in accomplishing the everyday and long-term objectives of QI in their organisations.

The interviews lasted between 60 and 90 min and were conducted via Skype or face-to-face by the lead author. All interviews were audio-recorded, transcribed verbatim and analysed using thematic analysis, a systematic approach to data coding and interpretation, utilising Nvivo® qualitative Analysis software [45, 46]. After initial inductive coding of the data and clustering of some codes by AA, the coding scheme was discussed with the two members of the research team (JF and RGr) and consensus on the codes was achieved. To compare the two groups of participants—traditional and clinical QIMs—some patterns related to each group were identified using the within-group coding approach [47]. Initial codes were then aggregated in three emergent themes. These themes were then provided to the participants for a member check to ensure the accuracy of interpretation and improve the validity of the study [48, 49]. When participants disagreed with the researchers’ interpretations, follow-up interviews were arranged to clarify the points of disagreement and the relevant changes to interpretations were made.

The consolidated criteria for reporting qualitative research (COREQ) were used to inform reporting of the study findings (Supplementary File 2) [50].

### Patient and public involvement

Patients or members of the general public were not involved in the design or conduct of this study.

## Results

We interviewed 56 QIMs from 15 NZ DHBs. Participant information is shown in Table 1. We identified three themes: QI expertise, leadership competencies and interpersonal competencies. We separately present (Supplementary File 3) the coding scheme in detail including the major themes (1^st^ order codes), sub-themes (2^nd^, 3^rd^ and 4^th^ order codes) and the number of participants from each group that cited them as perceived characteristics of successful QIMs.

### QI Expertise

Within this theme participants mentioned a variety of expertise-related topics including formal training, knowledge and experience in the use of specific QI methodologies and tools as well as experience of work in healthcare and awareness of this context.

#### QI knowledge and experience

Expertise in QI was often mentioned as essential for disseminating QI principles—frameworks, tools and good practices with traditional QIMs being the most vocal stressing the role of QIMs’ expertise in establishing credibility and legitimacy of QI in healthcare:

You need to be an expert in what you are preaching. Whatever QI method it is, you need to be an expert so that when people come to you, you can solve their problem.

**Participant 38**

The early-career QIMs (with experience of two to five years) reported continuous learning as a part of their work experience. They noted that the QI training they receive tends to focus on the theory, which primarily explores the use of QI tools and techniques, but when the QIMs begin using the QI tools in their actual work in healthcare, they face difficulties implementing the changes. Consequently, the QI training does not prepare them for handling and dealing with adverse incidents that arise during the implementations, requiring further on-the-job learning:

After learning QI, when I came back [to my DHB] and did my first project, I thought naively that everything will work out as they taught me but the real world is more dynamic. So, the first project went well but I made so many mistakes. I have learned my lesson and I am still learning new ones every day.

**Participant 6**

#### Healthcare Specific Experience

In addition to expertise in QI methodologies, QIMs mentioned the need for past work experience in healthcare sector specifically. This experience was viewed as enabling QIMs’ legitimacy by demonstrating familiarity with the functioning of the healthcare system and its differences from other sectors and industries [28]. Traditional QIMs who joined their DHBs after working in manufacturing—related industries primarily—or even service industries other than healthcare—mentioned the various difficulties they faced related to the lack of trust from other healthcare employees when they started their career in healthcare. The primary reason for this distrust from their colleagues was based on their lack of experience in healthcare sector and perceived poor understanding of its needs and values. The importance of the healthcare expertise was mostly underscored by the clinical QIMs not experiencing such difficulties in their work:

I think it's one of those outsider problems. It was a big thing in the past when QI was very new. I don’t think it is as bad nowadays. I don’t think nurses or physicians like me face it because we are from this system.

**Participant 12**

### Leadership competencies

Our data suggested that the QIM’s job is not just to be well-versed in the QI tools and their implementation; their job was to promote, stimulate and support the QI culture in their DHBs, essentially fulfilling the leadership role. The leadership competencies mentioned by participants include:

*Sense-giving*: Sense-giving is “concerned with the process of attempting to influence the sensemaking and meaning construction of others toward a preferred redefinition of organizational reality” [51]. It is critical in change management exercises such as QI initiatives as it ensures that all the stakeholders have the same vision for the changed organisation and they strive to attain the same goals.

As mentioned earlier, QI was developed in the manufacturing sector and, hence, it is often viewed as a foreign agenda in healthcare. QIMs were therefore found to play an important role in helping their colleagues to make sense of QI and its relevance and importance for their everyday work of care. This sense-giving exercise, with respect to QI, becomes especially important in the multi-stakeholder multi-logic context of healthcare [28], when different stakeholders hold unique worldviews and differently interpret organisational goals and objectives:

Sense-giving is crucial in QI. QI is everyone’s job and people don’t see it like that. I think our job is to ensure that this thinking that QI is everyone’s job only then can you have a sustainable QI initiative in a DHB.

**Participant 4**

#### Long-term Thinking

Participants agreed that successful QIMs adopt a long-term view of quality and build the capacity and capability of healthcare employees to undertake their own QI activities. QIMs assume a facilitative rather than directive approach and take a proactive view to implementation. Participants noted that the absence of this QIM’s competence may result in significant challenges and inefficiencies in QI implementation:

I have a simplistic understanding because my training was six months in total. That’s the same for other nurses in my team. I can do small initiatives and mostly, they won't work either because I don’t have this skill. But my DHB thinks that’s enough to ensure a long-term QI agenda. It's ridiculous.

**Participant 9**

#### Systems-thinking—an extended view of the HO

The work of QIMs necessitated that QI was understood in the context of the whole HO to avoid ‘sub-optimisation’ with improvement in one department or function leading to difficulties in other parts of the HO. Without this ‘whole of system’ view, QIMs adopted a myopic or narrow view of QI:

If I think about it, my position in the organisational structure is very appropriate for the job. I am well connected to everything vertically and horizontally. In my previous job [in another DHB], I lacked that and it made things very hard.

**Participant 10**

#### Motivating

Leadership competencies included the ability to motivate colleagues and direct their efforts. QIMs required *reward power* to motivate healthcare employees to engage in QI given that such work was regarded by many healthcare employees as secondary to clinical activities. Rewards were not necessarily monetary. In fact, participants noted that non-monetary rewards such as being recognised by senior management or receiving a national QI award were more effective than monetary rewards. A possible reason is that monetary rewards hold a negative connotation in the minds of both healthcare employees and QIMs. In the latter case, QIMs were concerned that healthcare employees would only participate in QI activities if given monetary rewards:

I personally think that monetary rewards are not going to be useful in the long-term. We don’t give monetary rewards because they will set a very wrong precedent. We don’t want them to do QI just for money.

**Participant 24**

Similarly to expertise, when clinical and traditional QIMs attributed different importance to the same competence, leadership competencies were highlighted by the traditional QIMs more than the clinical QIMs. This may be because the scope of the influence of the clinical QIMs was smaller, mostly restricted within a ward/department. We had clinical QIMs from multiple DHBs who kept their focus on nursing operations, primarily because these QIMs were nursing staff themselves and they did not recognise other operational and organisational areas to have issues with quality:

We are mainly focused on nursing operations in my DHB because it is very crucial for us… Other places require improvement, but I am not familiar with them right now. Maybe once we are done here, we can move to other parts of the DHB too.

Participant 30

When clinical QIMs were from particular wards, they lacked the extended view of the organisation, and therefore, they did not focus on the systems thinking element of QI. This prevented them from conducting transformational initiatives at an organisational level:

My job is to make sure renal ward [my ward] is doing well. So, all my initiatives deal with only renal ward. I guess somethings might affect others but, that is outside the boundaries of my work.

**Participant 11**

### Interpersonal Competencies

 Participants suggested that effective QIMs are required to have good interpersonal competencies, which include approachability, supportiveness and trustworthiness.

#### Approachability

The QIMs should be *approachable* by other employees so that employees do not hesitate to put forward ideas for improvements and QI or to call for any help and assistance. This also means creating a low power distance between the QIMs and the frontline employees to reduce any potential for hesitancy. To establish relationships, traditional QIMs mentioned using feedback loops and suggestions between themselves and the employees as an effective practice. They explained that the frontline employees are closer to the day-to-day work and the underlying operational processes, and hence, they have a better vantage point to recognise areas for improvements and value-adding processes and the inefficiencies in the healthcare system itself.

#### Supportiveness

In addition, QIMs were required to be *supportive* given most healthcare employees lack QI expertise and require ongoing support (which is not sustainable long-term). The QIMs conducted training activities to develop healthcare employee capabilities in QI, a strategy that depended on healthcare employees feeling empowered and supported by the QIMs:

QI requires a two-way relationship with the staff. I help them out, they help me out. We both need to support each other. If I am not supporting them and seeking their support only, I am not an effective QI manager.

**Participant 5**

#### Trustworthiness

Finally, effective QIMs are *able to build trust with the* employees in the organisation. If they are not trusted by the healthcare employees, they tend not to approach the QIMs with their problems. Participants mentioned that it takes time to build trust between QIMs and healthcare employees. Ideally, QI initiatives provide benefits to both QIMs and healthcare employees which then develops trust in QI and QIMs suggestions. To build trust, QIMs also promoted the notion that healthcare employees and QIMs were on the same ‘team’ (rather than two stakeholders with opposing interests). However, this strategy required reducing the power distance between QIMs and healthcare employees as described earlier. Taken together, interpersonal competencies help QIMs to introduce, get ‘buy-in’ and ensure the sustainability of QI initiatives.

Once again, the interpersonal competencies were perceived to be more important for traditional QIMs as they mentioned them to be useful far more times than the clinical group. These competencies are necessary to increase traditional QIMs’ legitimacy and mitigate the ‘outsider effect’. Trust and ability to establish and support a two-way communication are also important to gain support for initiatives that were developed outside the healthcare and could be seen as contradicting a healthcare logic.

Table 2 provides further detail regarding these characteristics, and the coding scheme including the major themes (1^st^ order codes), and sub-themes (2^nd^, 3^rd^ and 4^th^ order codes), and the number of participants from each group that cited them as perceived characteristics of successful QIMs.

**Table 2:** Summary of findings (Nvivo Coding)

Themes	QIMs (Clinical Staff)	QIMs (Operations/Process Engineers)
**QI Expertise**		
Experience of QI in healthcare	35/36	18/20
Implementing QI initiatives before	15/36	19/20
Understand healthcare operations	30/36	20/20
Understand healthcare issues	34/36	18/20
Care is the core value	35/36	16/20
Experience in QI (outside healthcare)	10/36	19/20
Qualifications in QI/operations management	11/36	20/20
Process engineering	5/36	20/20
Six Sigma certifications	5/36	18/20
Implementing QI initiatives before	9/36	19/20
Root cause problem solving experience	21/36	20/20
**Leadership Competencies**		
Sensegiving	22/36	20/20
Encourage people to adopt QI philosophy	20/36	20/20
Shows the positives of QI	22/36	20/20
Link QI objectives to personal values (care)	12/36	18/20
Long-Term Thinking	21/36	20/20
Long-term view	18/36	20/20
Explore unintended consequences of initiatives	10/36	18/20
Reward power	18/36	19/20
Motivation	25/36	18/20
Understand what motivates people	25/36	17/20
Be creative with incentives	10/36	18/20
Non-monetary incentives	15/36	15/20
Systems Thinking	15/36	19/20
Not part of a single department/division	12/36	19/20
Understand the needs of the complete organisation	14/36	18/20
Systems understanding	9/36	20/20
Link organisational silos and erase them	16/36	20/20
**Interpersonal Competencies**		
Approachability	20/36	18/20
Respects different opinions and views	27/36	17/20
Minimum power distance	22/36	15/20
Easy to talk to	20/36	16/20
Supportive	29/36	20/20
Coach others	15/36	20/20
Supports QI initiatives everywhere	8/36	20/20
Trustworthy	25/36	17/20
High trust among QIMs and frontline staff	24/36	17/20
Frontline staff sees them as ‘insider’, not ‘outsider’	18/36	20/20

## Discussion

### Summary of findings

This study provides a rich understanding of the core competencies and qualities of QIMs operating in NZ healthcare system. It sheds light on how QI can be facilitated in HOs by underscoring the core competencies and qualities of QIMs that are required in accomplishing the everyday and long-term objectives of QI in their HOs.

First, the study reveals that the QIMs largely associate their ability to successfully drive QI transformation in HOs with their personal competencies. Based on the interviews, three groups of competencies were identified: (1) expertise in QI which includes profound knowledge of QI methodologies and contextual competence (experience in healthcare); (2) leadership competencies comprising of long-term view, system thinking, sense-giving, and motivating abilities; and (3) interpersonal competencies represented by approachability, supportiveness and trustworthiness. These competencies allow QIMs to establish their legitimacy and lead the QI agenda with minimal resistance from the staff in their respective HOs.

Second, by differentiating between the two groups of QIMs, traditional and clinical QIMs, this study highlights the key challenges for both groups of QIMs, who often end up implementing QI in a ritualistic and superficial manner focusing on small pockets instead of a system-wide approach [10, 19–21, 52]. Indeed, traditional QIMs who bring with them extensive QI knowledge and skills often cannot implement initiatives in a meaningful way as they lack an understanding of the context of this implementation, the needs and expectations of various stakeholder groups as well as the ways to achieve their cooperation essentially lacking contextual and interpersonal competencies. Unless they become the *insiders* in the system, their efforts could be seen as ceremonial at best [28]. However, clinical QIMs are not necessarily in a better position. While being an *insider*—having internal knowledge and relationships required to get support for QI implementation—they overlook the need for such leadership competencies as systems thinking and long-term vision. Focusing on tools learnt during their short-term training, they implement QI at the unit-level, which often results in the transference of waste rather than eliminating it, impeding system-wide transformation [19, 21, 28].

### Comparison with existing literature

The existing literature undescores the importance of QIM competencies and suggests that different contexts may require different competence profiles from QIMs [53]. However, there is limited literature on QIMs in the health sector [29], and it has mostly focused on the QI implementations themselves rather than the QIMs’ role and expertise within these implementations [54]. Our study builds on previous attempts to understand the requirements to QIM competencies in healthcare [29, 30, 55]. It identifies the perspectives of the two key groups of healthcare QIMs, therefore, offering a refined understanding of key QIM competencies required in HOs.

While the QIM competencies mentioned in the findings are consistent with previous literature [29, 53–56], the importance and relationships between these competencies are specific to the healthcare context. Thus, it supports the literature in proposing that the QIMs’ competencies and their priority can be industry-dependent—a competency can be more crucial in a respective industry than the other. In particular, our study suggests that contextual competence, as raised in previous studies [34] (the expertise in healthcare environment and healthcare-specific knowledge), may be one of the key QIMs’ competence in healthcare [57, 58]. This finding emphasises that to successfully drive QI in healthcare, QIMs need to have strong associations and understand well this context; more importantly, they need to be viewed as the *insiders* by other employees [28]. Without this embeddedness in the health context, it is very difficult to carry out QI initiatives in a meaningful way [24] and ensure their sustainability [9, 59]. While clinical QIMs are already embedded in the healthcare context, traditional QIMs may require intensive support and socialisation.

Further, our findings underscore an important role of interpersonal competencies in the healthcare context and the role they play in facilitating the implementation of QI. This is not surprising, as interpersonal (human or communication) competencies were highlighted as important by previous studies separately [25, 29, 60, 61]. For example, the ‘house of’ competencies’ model developed by Ingason and Jónsdóttir put communication competencies at the top of the hierarchy [53], which is most important for QIMs. However, our study suggested that the relationship between interpersonal competencies of QIMs and their performance in healthcare might be more nuanced. Our participants highlighted that interpersonal competencies were important not on their own but in relation to other competencies and worked as the enablers of those competencies. Traditional QIMs strongly emphasised that, without these competencies, the potential of other competencies could be weakened (see Table 2). This again highlights that hierarchy and relationships among QIMs’ competencies may be different in different industries.

Perhaps unsurprisingly, being well-socialised in the healthcare environment, the clinical QIMs did not emphasise the role of interpersonal skills and local expertise in their success [62]. This suggests that clinical QIMs might be well positioned to carry out QI initiatives enjoying stronger trust, higher legitimacy and having a better understanding of the needs of healthcare. However, incumbent position may also come at some cost. Thus, we found that clinical QIMs did not pay much attention to driving change within their organisations which was reflected in the lack of mentioning of leadership competencies as necessary for successful QI implementation. The *insider* position and strong links with the sector perhaps prevents them from seeing the need for a radical change [63].

The development of leadership competencies is also not supported by the QI training for the clinical QIMs. One integral part of a QIM’s job is managing people across the HO and assisting them with QI initiatives. The training often provided to clinical QIMs in-house or within the healthcare sector is not necessarily comprehensive focusing on the application of QI tools to particular scenarios or situations. It lacks the exploration of QI as a systems improvement approach, characteristic of industrial QI training, thus not sufficiently developing such leadership competencies as long-term view and system thinking. This is why clinical QIMs often see QI as an ‘add-on’, rather than an integral part of the care process [20, 28, 64, 65]. This potentially translates into a ritualistic application of QI methods which seldom leads to better patient care even when significant healthcare resources are committed to QI activities [22, 25].

Overall, our study confirms the previous insights that QIM competence profiles need to be related to the contexts in which QIMs operate [53]. Furthermore, it suggests that in complex settings such as healthcare, different types of QIMs with different competence profiles may be required to fulfil a HO’s QI agenda.

### Limitations of the study

The data were collected only from the NZ healthcare system. Therefore, the findings regarding the QIMs may not be generalisable. While the NZ healthcare system closely resembles other national healthcare systems [66], and has constantly used and implemented similar QI programmes and training activities such as The Productive Ward, Releasing Time to Care and Institute for Healthcare Improvement’s Open School and Model for Improvement [67, 68], we still believe a future comparative multi-country study, focusing on a similar research question, could be valuable.

Second, while identifying QIMs’ account of the competencies and qualities they require in accomplishing the everyday and long-term objectives of QI in their organisations, this study does not test any relationship between and within these competencies and the actual QI performance of HOs. Therefore, further research seeking answers to the question of how competencies are related to each other and HO performance may be useful.

Interestingly, QIMs did not mention externally-oriented competencies—competencies, which are required for successful communication and interaction with external stakeholders. Given that HOs have multiple external stakeholders (e.g., patients, communities, governments etc.) interested in the improvement of health services quality, what are the implications of overlooking such relevant competencies by the QIMs? We believe that the context-specific competence profiles should pay special attention to these blind spots to understand why they appear and what are the implications of the absence of such competencies? Once again, a holistic approach to QI with a focus on co-design and co-production of health services may be a good start. Combining traditional QI with co-design can have significant outcomes [9, 69, 70].

Finally, the findings from our study hint at the difference in perspectives of the two groups of QIMs. Since both groups work in HOs and, moreover, quite often work together in the same team, it is important to understand the implications of their different views and perspectives for QI practice. It would be interesting and important to understand how these differences in perspectives influence the team dynamics and the development and implementation of the QI agenda in HOs. Future studies can look at how the diversity in QI teams could be leveraged to address the QI dilemma, which explores the balance between exploration and exploitation in QI [12, 71], and how the harmony between the different viewpoints could be achieved. Indeed, the medical professionalism logic based on patient care working along the managerial and QI logic of efficiency and continuous improvement tends to create tensions [28]. Therefore, understanding how these tensions are approached and resolved in a QI team could be of significant importance.

### Implications for practice and health policy

Our study differentiates between traditional QIMs and clinical QIMs suggesting how both of these groups can be better prepared and effective in their jobs. We believe that this study has crucial implications for practice. Both groups require a comprehensive socialisation and training process which should be designed to meet their specific needs, and HOs need to ensure such QIM needs are met so that their QI implementations have a higher rate of success.

For HOs, this study provides valuable practical insights. We believe that HOs need to provide better support for their QIMs, and ensure that they have the core competencies and qualities before they are required to conduct large-scale QI initiatives to improve their success rate. Thus, perhaps traditional QIMs can be educated to incorporate the concept of stakeholder value and multiple-stakeholder perspectives, a key feature of healthcare systems all around the world. More attention could be paid to the role of co-design methodologies in QI. In addition, traditional QIMs may require a comprehensive socialisation process when starting work in HOs with the emphasis on the understanding of the healthcare context. These approaches will enable traditional QIMs to better understand the context for QI and help them to gain support from different stakeholder groups within the HOs. Similarly, the training and development of the clinical QIMs should be widened and soft elements of QI—QI culture, philosophy and systems perspective—should be incorporated into it to provide them with the system perspective and prepare them to lead QI transformations. There is already some evidence to support our claims [12, 13]. However, more studies are required to understand the effects of holistic QI training, comprising of hard and soft QI tools, on QIMs and their effectiveness in HOs. Perhaps developing QI teams that comprise of both the traditional as well as clinical QIMs could be a good start. Research already suggests that cooperation among clinical staff and QIMs should help to decrease resistance towards QI in HOs [12, 28]. It should also help both of these groups to learn from each other and offset their weaknesses. However, further research into the efficacy of such QI teams is required.

## Conclusion

Healthcare has a long history of borrowing management strategies from other industries. This offers various lessons [72]. Indeed, after the advent of Lean Thinking in Toyota and Six Sigma in Motorola, other organisations in the industry replicated these strategies [73, 74]. However, these attempts mostly failed because the managers assigned to lead these transformations were unequipped and unprepared [75–79]. Therefore, it is important to understand how QIMs can be better prepared to perform their roles [29, 34]. While there is a plethora of research devoted to success factors in QI implementation at organisational level [80–84], surprisingly, research is scarce at the individual level [12]. This study adds to our knowledge by exploring traditional and clinical QIMs accounts of the competencies and qualities they require in accomplishing the everyday and long-term objectives of QI in their organisations. The findings can be used as a guide by HOs around the world to facilitate and better support both traditional and clinical QIMs in their organisational roles.

## Supplementary Information

**Additional file 1.**

**Additional file 2.**

**Additional file 3.**

## Data Availability

Full de-identified interview transcripts will not be shared. Informed consent, in line with the approving ethics committee, only allows for the use of de-identified extracts within research reporting and writing, in order to maintain the privacy of participants based in a defined regional area and population, thus making their identification with full transcripts more likely. For further inquiries, please contact the corresponding author.

## Abbreviations

QI: Quality Improvement
HO: Health Organisation
QIM: Quality Improvement Managers
NZ: New Zealand
DHB: District Health Boards
