# Supplementary material for: What makes an effective Quality Improvement Manager? A qualitative study in the New Zealand Health System
Source: BMC Health Serv Res. 2022 Jan 10;22:50. doi: 10.1186/s12913-021-07433-w (PMC8751312; doi:10.1186/s12913-021-07433-w)
Supplement: Supplementary file 1 — Additional file 1. [file 12913_2021_7433_MOESM1_ESM.docx]

# Theme 1: Understanding QI

(These questions are asked to primarily understand the knowledge of the participant regarding QI and their involvement in it)

1. As my research is about QI, before we start, would you be willing to tell me what QI means to you? What is it about?
2. What is your background? Did you have any formal QI or operations management training?
3. Are you a practicing clinician?
4. I read that the XXX DHB is implementing QI in the operations right now, to what extent are you or your office involved in it?
5. Do you see your involvement being crucial and necessary element in it?

# Theme 2: Need for QI

(These questions are asked to understand the reasons for why the QIM’s DHB decided to consider implementing QI; and where does the participant see themselves in this process? )

1. What were the main reasons/goals behind the decision for implementing QI here in the DHB?
2. How are these reasons/goals linked with the overall mission of the DHB as a healthcare providing organisation for the whole population?
3. Who were the main parties/entities who made the decision to implement it
   1. Did they have any prior experience with QI? Or were they briefed about it before the decision?
   2. Are they still involved with the QI implementation? To what extent?
4. How crucial was or is this QI implementation to the DHB?
5. What kind of value does it add to DHB as an organisation?
6. Are there any criteria in place to measure that value?

# Theme 3: Level of QI Maturity (QI Philosophy & Culture)

(*These questions are asked to primarily understand the maturity of QI in the organisation; and the participants’ point of regarding said maturity. The participants are probed to explain the challenges and other shortcomings in their organisations’ strategy and pathway to QI maturity*)

1. When did the organisation decided or started implementing QI in its’ operations?
2. Do you think QI is taken seriously in DHB?
3. What kind of audits, checks and monitoring criteria are there to ensure QI implementation is going smoothly?
   1. Which department deals with all of that?
4. Do you think a certain type of culture is required to implement and sustain QI implementations?
   1. What is that culture?
   2. Do you think your organisation has that culture?
5. Is management committed to QI implementation and promoting the QI culture?
6. How about employees’ commitment to promote/support the QI culture?
   1. What strategies are considered important in your organisation to engage staff in the QI project?
   2. How often do you get suggestions from employees related to QI implementation and initiatives in their job?
7. Does a name of a particular department come to your mind when you think about these suggestions or QI implementation in general?

# Theme 4: Level of QI Integration and QIMs responsibilities

*(These questions are asked to understand the level of QI integration and its’ extent in the organisation; and the QIMs responsibilities in improving the scope of QI).*

1. What is the scope of the QI implementation in your organisation?
2. Which departments are selected to be QI?
   1. What is the selection criteria for these chosen departments?
3. Is the focus more on support services or care providing departments?
4. Can you tell me about the QI implementation in supply chain?
5. Do you have QI in SC? Why or Why not?
6. How do suppliers react to it?
7. How do you motivate the suppliers to adopt QI?
8. Do you conduct any trainings for suppliers for QI?
9. Do you see any challenges regarding QI in supply chain?
10. What are/can you do to overcome them?
11. Are there any challenges you can’t do anything about?
12. Is there a particular entity responsible for the QI implementation process in the whole organisation?
13. Is there a particular entity responsible for the QI implementation in each department?
14. Are there any challenges that this entity faces with QI implementation in the DHB?
15. Do you see any certain strategies that work to mitigate these challenges?
16. How are these challenges being addreseed?
17. Are there any challenges that you can’t do anything about or can’t be mitigated?
18. Do you think you as a QIM have the right ammunition to drive QI agenda?
19. What’s missing?
20. Do you think the DHB is providing you enough support and resources to conduct your job?
21. What would you want more from the DHB in terms of the support and resources?
22. Are there any other DHBs that you see tackling these issues well?
23. What do they do about it?
