# Supplementary material for: What makes an effective Quality Improvement Manager? A qualitative study in the New Zealand Health System
Source: BMC Health Serv Res. 2022 Jan 10;22:50. doi: 10.1186/s12913-021-07433-w (PMC8751312; doi:10.1186/s12913-021-07433-w)
Supplement: Supplementary file 3 — Additional file 3. [file 12913_2021_7433_MOESM3_ESM.docx]

| **Themes** | **QIMs**  **(Clinical Staff)** | **QIMs**  **(Operations/Process Engineers)** |
| --- | --- | --- |
| **QI Expertise** |  |  |
| Experience of QI in healthcare | 35/36 | 18/20 |
| Implementing QI initiatives before | 15/36 | 19/20 |
| Understand healthcare operations | 30/36 | 20/20 |
| Understand healthcare issues | 34/36 | 18/20 |
| Care is the core value | 35/36 | 16/20 |
| Experience in QI (outside healthcare) | 10/36 | 19/20 |
| Qualifications in QI/operations management | 11/36 | 20/20 |
| Process engineering | 5/36 | 20/20 |
| Six Sigma certifications | 5/36 | 18/20 |
| Implementing QI initiatives before | 9/36 | 19/20 |
| Root cause problem solving experience | 21/36 | 20/20 |
| **Leadership Competencies** |  |  |
| Sensegiving | 22/36 | 20/20 |
| Encourage people to adopt QI philosophy | 20/36 | 20/20 |
| Shows the positives of QI | 22/36 | 20/20 |
| Link QI objectives to personal values (care) | 12/36 | 18/20 |
| Long-Term Thinking | 21/36 | 20/20 |
| Long-term view | 18/36 | 20/20 |
| Explore unintended consequences of initiatives | 10/36 | 18/20 |
| Reward power | 18/36 | 19/20 |
| Motivation | 25/36 | 18/20 |
| Understand what motivates people | 25/36 | 17/20 |
| Be creative with incentives | 10/36 | 18/20 |
| Non-monetary incentives | 15/36 | 15/20 |
| Systems Thinking | 15/36 | 19/20 |
| Not part of a single department/division | 12/36 | 19/20 |
| Understand the needs of the complete organisation | 14/36 | 18/20 |
| Systems understanding | 9/36 | 20/20 |
| Link organisational silos and erase them | 16/36 | 20/20 |
| **Interpersonal Competencies** |  |  |
| Approachability | 20/36 | 18/20 |
| Respects different opinions and views | 27/36 | 17/20 |
| Minimum power distance | 22/36 | 15/20 |
| Easy to talk to | 20/36 | 16/20 |
| Supportive | 29/36 | 20/20 |
| Coach others | 15/36 | 20/20 |
| Supports QI initiatives everywhere | 8/36 | 20/20 |
| Trustworthy | 25/36 | 17/20 |
| High trust among QIMs and frontline staff | 24/36 | 17/20 |
| Frontline staff sees them as ‘insider’, not ‘outsider’ | 18/36 | 20/20 |
